# Niclosamide Is Active In Vitro against Mycetoma Pathogens

**DOI:** 10.3390/molecules26134005

**Published:** 2021-06-30

**Authors:** Abdelhalim B. Mahmoud, Shereen Abd Algaffar, Wendy van de Sande, Sami Khalid, Marcel Kaiser, Pascal Mäser

**Affiliations:** 1 Department of Medical Parasitology and Infection Biology, Swiss Tropical and Public Health Institute, 4051 Basel, Switzerland; halim.mahmoud@swisstph.ch (A.B.M.); marcel.kaiser@swisstph.ch (M.K.); 2 Faculty of Science, University of Basel, 4001 Basel, Switzerland; 3 Faculty of Pharmacy, University of Khartoum, Khartoum 11111, Sudan; 4 Faculty of Pharmacy, University of Science and Technology, Omdurman 14411, Sudan; phd_sh086@hotmail.com (S.A.A.);; 5 Erasmus Medical Center, Department of Medical Microbiology and Infectious Diseases, 3000 Rotterdam, The Netherlands; w.vandesande@erasmusmc.nl

**Keywords:** mycetoma, *Madurella mycetomatis*, *Actinomadura*, drug repurposing, nitroimidazole, salicylanilide, niclosamide, MMV665807

## Abstract

Redox-active drugs are the mainstay of parasite chemotherapy. To assess their repurposing potential for eumycetoma, we have tested a set of nitroheterocycles and peroxides in vitro against two isolates of *Madurella mycetomatis*, the main causative agent of eumycetoma in Sudan. All the tested compounds were inactive except for niclosamide, which had minimal inhibitory concentrations of around 1 µg/mL. Further tests with niclosamide and niclosamide ethanolamine demonstrated in vitro activity not only against *M. mycetomatis* but also against *Actinomadura* spp., causative agents of actinomycetoma, with minimal inhibitory concentrations below 1 µg/mL. The experimental compound MMV665807, a related salicylanilide without a nitro group, was as active as niclosamide, indicating that the antimycetomal action of niclosamide is independent of its redox chemistry (which is in agreement with the complete lack of activity in all other nitroheterocyclic drugs tested). Based on these results, we propose to further evaluate the salicylanilides, niclosamide in particular, as drug repurposing candidates for mycetoma.

## 1. Introduction

First described from Madurai as Madura foot, the disease eumycetoma is endemic in the ‘mycetoma belt’ from India to the Middle East and across the Sahel [1,2]. The highest prevalence today is in Sudan [1,2]. Eumycetoma is a chronic subcutaneous mycosis that slowly spreads, starting from an initial lesion at the site of inoculation into the skin and deeper tissues, ultimately destroying muscles, tendons, and bones. The leg and foot are most often affected, likely due to inoculation via thorn pricks. While eumycetoma can be caused by various fungi of the orders Sordariales and Pleosporales [3], the majority of cases in Sudan are due to *Madurella mycetomatis* [2]. Eumycetoma is a debilitating, disfiguring, and stigmatizing disease. It is also an enigmatic disease in the light of the many open questions regarding its epidemiology, pathogenesis, and the biology of the causative agents [2,4].

In contrast to actinomycetoma, which is caused by filamentous bacteria and can be treated with antibiotics, there is no satisfactory treatment for eumycetoma. The current therapy consists of a combination of surgery and long-term chemotherapy with antifungal azoles, such as itraconazole [2]. However, the cure rates are low, and amputation of the affected limb may be the only measure to stop the flesh-eating fungus [5]. Given the urgent need for better drugs and the fact that eumycetoma is a neglected disease affecting neglected patients, drug repurposing suggests itself as a fast and cost-effective way towards new antimycetomal agents [6,7].

In this article, we pursued this strategy by testing a small set of redox-active parasiticides and antibiotics for their in vitro activity against *M. mycetomatis*. Redox-active molecules are the mainstay of current parasite chemotherapy [8]. The artemisinins for malaria, benznidazole and nifurtimox for Chagas’ disease, fexinidazole for sleeping sickness, and metronidazole for intestinal protozoa: all of these are prodrugs that will produce cytotoxic radicals once they are activated by the parasite’s metabolism. The activation occurs by chemical reduction, i.e., the acquisition of an electron [9]. Since saprophytic fungi dwell in hypoxic environments and may possess reducing agents of low redox potential, we speculated that *Madurella*, too, might be susceptible to prodrugs that are activated by electron transfer.

## 2. Results

**Figure 1:**
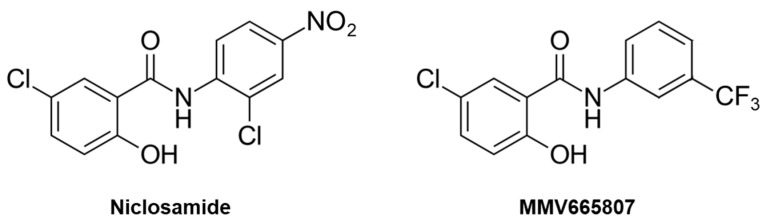
Chemical structure of niclosamide and MMV665807: Both compounds are salicylanilides, i.e., amides of salicylic acid and aniline.

A selection of redox-active drugs and experimental compounds was evaluated for their in vitro activity against two different isolates of *M. mycetomatis*, SO1 and CBS131320. It included six nitroimidazoles (fexinidazole, metronidazole, secnidazole, plus three experimental compounds [10,11]), three nitrofurans (nifurtimox, nifuroxacide, nitrofurantoine), a salicylanilide (niclosamide), and five peroxides (artemisinin, dihydroartemisinin, artesunate, artemether, and the experimental ozonide OZ78). Itraconazole was included as a reference. All molecules were tested in serial dilution against *M. mycetomatis* cultures [12]. The minimal inhibitory concentration (MIC) was defined as the lowest concentration that, after 7 days of incubation, had inhibited the growth by at least 80% compared to untreated cultures. Itraconazole had a MIC of 0.13 µg/mL and 0.25 µg/mL against SO1 and CBS131320, respectively (Table 1). The tested peroxides were inactive, which is in agreement with the reported lack of activity of artemisinin [13]. Moreover, all the nitroimidazoles and nitrofurans were inactive. The one notable exception was niclosamide (Figure 1), which had a MIC around 1 µg/mL (Table 1).

This interesting finding was followed up by testing the three compounds niclosamide, niclosamide-ethanolamine (NEN), and MMV665807 against the two isolates of *M. mycetomatis* and two species of *Actinomadura*, *A. madurae* and *A. syzygii*, causative agents of actinomycetoma. NEN is the ethanolamine salt of niclosamide [14]. MMV665807 (Figure 1) is a salicylanilide from the Medicines for Malaria Venture’s malaria box [15] that has shown antibacterial [16], antiprotozoal [17,18], and anticestodal [19,20] activity. All three compounds exhibited good activity against *Madurella* as well as *Actinomadura*, with MIC values somewhat higher than the reference drug itraconazole for *M. mycetomatis* and considerably lower than the reference drug cotrimoxazole for *Actinomadura* (Table 2).

## 3. Discussion

In this small drug repurposing study, we have evaluated redox-active parasiticides and antibiotics against *M. mycetomatis* based on the rationale that the metabolism of the fungus would be able to reduce, and thereby activate, the prodrugs. This hypothesis turned out to be wrong, as all tested molecules were inactive—except for niclosamide. Given the lack of activity of the tested nitrofurans and nitroimidazoles, the observed activity of niclosamide is likely not due to its nitro group, but rather due to the salicylanilide moiety. This is supported by the good activity of MMV665807 against *M. mycetomatis*, a salicylanilide that lacks a nitro group (Figure 1). Thus, the discovery of niclosamide as a hit for mycetoma pathogens was serendipitous. The nitro group of niclosamide was found to be dispensable also for its inhibitory action on Wnt signaling, an activity that has raised interest in niclosamide as an anticancer agent [21]; another such activity is mitochondrial uncoupling [22,23].

Niclosamide is an old drug of many uses [24,25]. It was developed by Bayer (Bayer 2353) in the 1950s as a molluscicide for schistosomiasis control. Since 1982, when it was approved by the FDA for human use, its primary indication has been as a broad-spectrum anthelmintic for tapeworms (*Taenia* spp., *Diphyllobothrium latum*) and intestinal fluke (*Fasciolopsis buski*) [26]. Niclosamide was shown to have promising activity against bacteria [27,28], fungi [29], and even coronavirus [30,31]. What restricted its use to intestinal pathogens was the poor oral bioavailability, i.e., the fact that niclosamide is not significantly absorbed from the gastrointestinal tract [32,33]. Different carriers or formulations have been employed to overcome this issue, for example, [34,35,36]. The ethanolamine salt of niclosamide (NEN, also called niclosamide olamine or clonitralide) has a better water-solubility and bioavailability [33], and it is being considered for different (re)purposes [22,37,38,39,40]. In our in vitro assays, NEN was as active as niclosamide against *M. mycetomatis* and *Actinomadura* spp.

The finding that a drug like niclosamide, which is on the WHO’s list of Essential Medicines, exhibited in vitro activity against both *Madurella mycetomatis* and *Actinomadura* spp. in the same range as the reference compounds, warrants the testing of further salicylanilides against these pathogens and the consideration of niclosamide as a repurposing candidate for mycetoma.

## 4. Materials and Methods

### 4.1. Chemicals

MMV665807 (Princeton Bio Molecular Research) and niclosamide-ethanolamine (2A Biotech) were received from Britta Lundström-Stadelmann (University of Bern, Switzerland), the five peroxides from Jonathan Vennerstrom (University of Nebraska, USA). All other test compounds were obtained from DNDi (Geneva, Switzerland). Ro 15-6547 is 4′[(1-methyl-2-nitroimidazole-5-yl)methoxy]-1-pyrrolidine-acetanilide, and RJ-55 and RJ-164 are 1-aryl-4-nitroimidazoles; the three nitroimidazoles were developed for African trypanosomiasis [10,11]. OZ78 is an ozonide related to arterolane [41,42] that has mainly been studied for its activity against trematodes [43,44,45]. All compounds were dissolved in DMSO.

### 4.2. Strains

*Madurella mycetomatis* isolate SO1 was originally isolated from a Somalian patient and CBS131320 from a Sudanese patient. The two PCR-identified strains were obtained from the mycetoma collection of the Erasmus Medical Centre, Rotterdam, The Netherlands. The mycelia were grown at 37 °C in RPMI 1640 medium supplemented with 0.35 g/L l-glutamine and 1.98 mM 4-morpholinepropane sulfonic acid. *Actinomadura madurae* SAK-A05 and *Actinomadura syzygii* SAK-A08 were originally isolated from Sudanese patients and grown in the pharmaceutical research laboratory, University of Science and Technology repository (Omdurman, Sudan). The bacterial suspensions were prepared in Mueller Hinton II broth (CAMHB) media and propagated at 35 °C.

### 4.3. In Vitro Drug Efficacy Testing

*M. mycetomatis* mycelia in RPMI 1640 medium were sonicated for 10 s (QSONICA Q55) and centrifuged at 2600× *g* for 5 min. The mycelia were washed and resuspended in fresh RPMI 1640 medium to obtain a fungal suspension of 68% to 72% transmission at 660 nm (UV/Vis Spectrophotometer 6305, Jenway, UK). A 1:2 serial drug dilution covering a range from 256 μg/mL to 0.063 μg/mL of test compound was prepared in a round-bottom 96-well microtiter plate (Corning, CLS379). Itraconazole was used as the positive control at a range from 1 μg/mL to 0.03 μg/mL. Exactly 100 µL of adjusted fungal suspension was added to each well along with 1 µL of the drug. Next, 1 µL of resazurin was added to a final concentration of 0.15 mg/mL [12]. The plates were sealed and incubated at 37 °C for 7 days. All assays were performed in triplicate. For further details see [12].

*Actinomadura* suspensions were adjusted to absorbance of 0.08–0.1 at 625 nm. A 1:2 serial drug dilution covering a range from 256 μg/mL to 0.063 μg/mL was prepared in a 96-well microtiter plate. Co-trimoxazole (fixed combination of 1 part trimethoprim and 5 parts sulfamethoxazole) was employed as the positive control at a starting concentration of 80 µg/mL. Next, 100 µL of adjusted bacterial suspension were added to each well along with 1 µL of the drug. Precisely 1 µL of resazurin was added to a final concentration of 0.15 mg/mL. The plates were incubated at 35 °C for 5 days. All assays were performed in triplicate.

The assay plates were inspected for visual and spectrometric endpoints. Absorbance was measured at 600 nm (Thermo Scientific Multiskan Spectrum, Thermo Fisher Scien-tific, Vantaa, Finland). The minimal inhibitory concentration (MIC) was defined as the lowest concentration of test compound that produced ≥80% growth inhibition as compared to the untreated cultures (negative control).

## Figures and Tables

**Table 1:** Selected redox-active agents and their in vitro activity against *M. mycetomatis*: All assays were performed in triplicate. Values are minimal inhibitory concentrations (MIC) in µg/mL.

Compound	Class	Indication ^1^	SO1 ^2^	CBS131320 ^2^
Niclosamide	Salicylanilide	Tapeworms	0.78	1.6
Secnidazole	Nitroimidazole	Bacterial vaginosis	>256	>256
Metronidazole	Nitroimidazole	Broad spectrum antibiotic	>256	>256
Fexinidazole	Nitroimidazole	Human African trypanosomiasis	>256	>256
RJ-164	Nitroimidazole	(Human African trypanosomiasis)	>256	>256
RJ-55	Nitroimidazole	(Human African trypanosomiasis)	>256	>256
Ro 15-6547	Nitroimidazole	(Human African trypanosomiasis)	>256	>256
Nifurtimox	Nitrofuran	Chagas’ disease, HAT	>256	>256
Nifuroxazide	Nitrofuran	Colitis and diarrhea	>256	>256
Nitrofurantoine	Nitrofuran	Urinary tract infections	>256	>256
OZ 78	Peroxide	(Malaria, trematodes)	>256	>256
Artemisinin	Peroxide	Malaria	16	16
Dihydroartemisinin	Peroxide	Malaria	>256	>256
Artesunate	Peroxide	Malaria	>256	>256
Artemether	Peroxide	Malaria	64	64
Itraconazole	Triazole	Antifungal	0.13	0.25

^1^ For experimental compounds, the envisaged indication is in parentheses; ^2^ SO1 and CBS131320 are two different isolates of *M. mycetomatis*.

**Table 2:** In vitro activity of niclosamide and related compounds against causative agents of mycetoma: All assays were performed in triplicate. Values are MIC in µg/mL.

Compound	SO1 ^1^	CBS131320 ^1^	SAK-A05 ^2^	SAK-A08 ^2^
Niclosamide	0.78	1.6	0.39	0.39
Niclosamide-ethanolamine	0.78	1.6	0.19	0.39
MMV665807	1.6	1.6	0.39	0.39
Itraconazole	0.13	0.25	n.d.	n.d.
Cotrimoxazole	n.d.	n.d.	20	10

^1^ SO1 and CBS131320 are two different isolates of *M. mycetomatis*.^2^ SAK-A05 and SAK-A08 are two species of *Actinomadura* (*A. madurae* and *A. syzygii*, respectively).

## Data Availability

All data are contained within the article.

## Sample Availability

For samples of the compounds please contact the corresponding author, who will link you up with the compound providers (see Acknowledgments above).
